# Reducing Lissencephaly-1 levels augments mitochondrial transport and has a protective effect in adult *Drosophila* neurons

**DOI:** 10.1242/jcs.179184

**Published:** 2016-01-01

**Authors:** Alessio Vagnoni, Patrick C. Hoffmann, Simon L. Bullock

**Affiliations:** Division of Cell Biology, MRC Laboratory of Molecular Biology, Francis Crick Avenue, Cambridge CB2 0QH, UK

**Keywords:** Axonal transport, Mitochondria, Lissencephaly-1, *Drosophila*

## Abstract

Defective transport of mitochondria in axons is implicated in the pathogenesis of several age-associated neurodegenerative diseases. However, the regulation and function of axonal mitochondrial motility during normal ageing is poorly understood. Here, we use novel imaging procedures to characterise axonal transport of these organelles in the adult *Drosophila* wing nerve. During early adult life there is a boost and progressive decline in the proportion of mitochondria that are motile, which is not due to general changes in cargo transport. Experimental inhibition of the mitochondrial transport machinery specifically in adulthood accelerates the appearance of focal protein accumulations in ageing axons, which is suggestive of defects in protein homeostasis. Unexpectedly, lowering levels of Lissencephaly-1 (Lis1), a dynein motor co-factor, augments axonal mitochondrial transport in ageing wing neurons. *Lis1* mutations suppress focal protein accumulations in ageing neurons, including those caused by interfering with the mitochondrial transport machinery. Our data provide new insights into the dynamics of mitochondrial motility in adult neurons *in vivo*, identify Lis1 as a negative regulator of transport of these organelles, and provide evidence of a link between mitochondrial movement and neuronal protein homeostasis.

## Abstract (precis)

**Summary:** Novel imaging procedures in the adult *Drosophila* wing reveal that Lissencephaly-1 restrains mitochondrial motion and that reducing levels of this protein protects against an age-related decline in protein homeostasis.

## INTRODUCTION

Distribution of vesicles and organelles by cytoskeletal motors plays a key role in neuronal function (Millecamps and Julien, 2013). The importance of cytoskeletal transport is underscored by the discovery of causative mutations in microtubule motor proteins and their co-factors in several human neurological disorders (Bi et al., 2009; Lipka et al., 2013; Puls et al., 2003; Reid et al., 2002; Vissers et al., 2010; Yamada et al., 2003; Zhao et al., 2001).

Mitochondria have a central role in cellular homeostasis, with roles including the production of ATP and reactive oxygen species, buffering levels of Ca^2+^ ions and regulating apoptosis. Defective transport of these organelles has been implicated in the pathogenesis of several age-associated neurodegenerative diseases. Pathologically modified proteins that cause amyotrophic lateral sclerosis (ALS) (De Vos et al., 2007), Charcot–Marie–Tooth disease (Misko et al., 2012), Parkinson's disease (Godena et al., 2014), hereditary spastic paraplegia (Kasher et al., 2009), Alzheimer's disease (Shahpasand et al., 2012) and Huntington's disease (Orr et al., 2008) can inhibit mitochondrial transport in cultured mammalian neurons. Impaired axonal transport of mitochondria has also been observed in a pre-symptomatic stage in a mouse model of ALS (Bilsland et al., 2010; Marinkovic et al., 2012). However, it is unclear whether defective mitochondrial transport has a causative role in neurodegenerative disease or is a consequence of an already compromised cellular state. Nonetheless, it has been suggested that interventions that increase transport of mitochondria might ameliorate neuronal dysfunction in a disease context (Hinckelmann et al., 2013). This hypothesis has been difficult to test, in part because few strategies are available for boosting transport of these organelles.

Several groups have shown that trafficking of mitochondria also strongly influences the neurodegenerative response to axonal injury. Intriguingly, some studies have provided evidence that axonal mitochondria are needed for neurodegeneration following injury (Barrientos et al., 2011; Keller et al., 2011), whereas others support a protective role for these organelles in this context (Avery et al., 2012; Fang et al., 2012, 2014; Ohno et al., 2014; Rawson et al., 2014).

Although much attention has focused on the links between axonal mitochondrial transport and neurodegeneration in a disease or injury setting, the regulation of mitochondrial movement during normal ageing of neurons *in vivo* is poorly understood. Studying this process requires imaging of cargo dynamics in live animals. Currently available methods for intravital imaging of axonal transport in vertebrate model organisms are technically challenging (Misgeld et al., 2007; Plucinska et al., 2012). Moreover, longitudinal studies of mitochondrial transport are very time-consuming in these animals because of the lifespan of the organism (Milde et al., 2015).

Here, we demonstrate that the wing marginal nerve of the fruit fly *Drosophila melanogaster* is a tractable system for detailed analysis of organelle transport in ageing adult neurons. We reveal striking changes in mitochondrial motion over time and identify Lissencephaly-1 (Lis1) as a negative regulator of initiation of mitochondrial transport. Moreover, we provide evidence of a link between movement of mitochondria and protection against focal protein accumulations in ageing axons.

## RESULTS

### Characterisation of axonal transport of mitochondria in wing sensory neurons

**Fig. 1.**
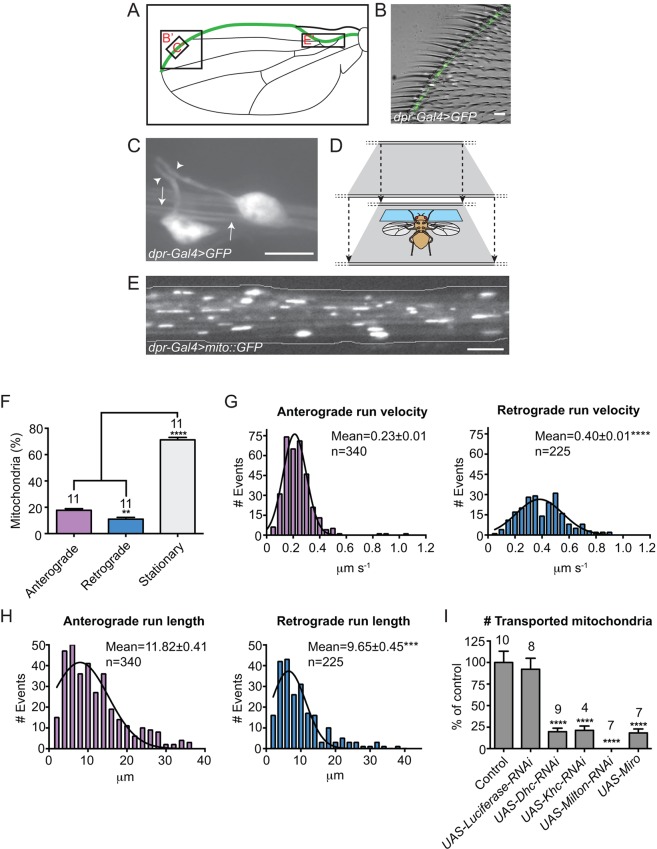
**Characterisation of mitochondrial transport in the wing nerve.** (A) Cartoon of the *Drosophila* wing, with the position of the wing nerve highlighted in green. Boxes B′, C′ and E′ show regions imaged for panels B, C and E. (B) Combined fluorescence and bright-field confocal image of the wing margin of a *dpr-Gal4 UAS-GFP* fly, in which chemosensory neurons are labelled fluorescently. (C) High-magnification image of wing neuron cell bodies and processes. Arrowheads, dendrites; arrows, bundled axons. (D) Schematic of the chamber used for imaging of organelle transport in the wing nerve. Blue rectangle, double-sided tape used to mount the fly. Wings are mounted in halocarbon oil. (E) Still of GFP-labelled mitochondria in axons in the wing arch region 1 day after eclosion. (F–H) Quantification of motile properties of mitochondria in the wing arch of *dpr-Gal4 UAS-mito::GFP* flies at 1 day after eclosion. Data are expressed as mean±s.e.m. In G and H, black lines indicate fitted curve; mean±s.e.m. values per run were calculated from raw values (*n*, number of runs), with statistical significance evaluated compared to the same parameter in the anterograde direction. (I) Quantification of relative number of transported mitochondria in the wing arch at 2 days after eclosion. *Dhc, Dynein heavy chain*; *Khc*, *kinesin-1 heavy chain*. Mitochondria were marked with *dpr-Gal4 UAS-mito::GFP*. The control genotype is *dpr-Gal4 UAS-mito::GFP* only. In F–I, each movie was captured for 3 min. In F and I, the numbers of wings analysed are shown above the bars. **P<0.01; ***P<0.001; ****P<0.0001 [one-way ANOVA with Holm–Sidak's multiple comparison test (F,I) or two-tailed Student's *t*-test (G,H)]. Scale bars: 5 µm.

The wing marginal nerve of *Drosophila* is located at the anterior margin of the wing (Fig. 1A,B) and comprises chemosensory (Fang et al., 2012; Nakamura et al., 2002) and mechanosensory (Palka et al., 1979; Fig. S1A–C) neurons. The cell bodies of these neurons are connected to the bristles by short dendrites (Fig. 1C), and to the thoracic ganglion of the central nervous system by long axons that bundle together and project through the wing arch. These axons are well suited for light microscopy studies because of the accessibility and translucent nature of the wing. Previous cell biological studies of the wing nerve have focused on axonal injury, with fluorescent proteins imaged with low spatial and temporal resolution (Fang et al., 2012, 2013; Neukomm et al., 2014; Soares et al., 2014). We developed new procedures for mounting and imaging wings of live animals that allow neuronal protein dynamics to be followed in detail (Fig. 1D,E; Fig. S1A–C; Materials and Methods).

We took advantage of the binary UAS-Gal4 system (Brand and Perrimon, 1993) to express a GFP-tagged marker of the mitochondrial matrix (mito::GFP; Pilling et al., 2006) in the wing nerve (Fig. 1E). For these experiments, we used a *UAS-mito::GFP* transgene and the *dpr-Gal4* driver, which is active in chemosensory neurons throughout developmental and adult stages (Fang et al., 2012; our unpublished observations). We initially filmed the wing arch of flies 24 h after eclosion from the pupal case. Approximately 30% of mitochondria exhibited bouts of directional transport during a 3-min period of filming, with the remainder stationary for the entire time (Fig. 1F; Movie 1). This proportion of motile mitochondria is similar to that documented in other systems, including in motor neurons in *Drosophila* larvae and the sciatic nerve of mice (MacAskill and Kittler, 2010; Misgeld et al., 2007; Pilling et al., 2006). Anterograde runs were more common than retrograde runs in wing axons (Fig. 1F), with individual organelles hardly ever switching directions. Manual tracking of transported mitochondria revealed that they underwent long bouts of transport in each direction with velocities of up to 1 μm/s (Fig. 1G,H), which is also consistent with observations in other neurons (MacAskill and Kittler, 2010; Misgeld et al., 2007; Pilling et al., 2006). No significant differences in the motile properties of mitochondria were observed between male and female flies (Table S1).

We next evaluated the effects of inhibiting known components of mitochondrial transport machinery on transport in the wing nerve. We targeted the microtubule motors cytoplasmic dynein-1 (dynein) and kinesin-1, which translocate mitochondria towards the minus and plus ends of microtubules, respectively, and the mitochondrial motor adaptor proteins Miro and Milton (TRAK1 and TRAK2 in vertebrates) (Saxton and Hollenbeck, 2012). Expression of RNA interference (*UAS-RNAi*) constructs that target the dynein or kinesin-1 heavy chain subunits with *dpr-Gal4* strongly reduced the number of mitochondria that underwent axonal transport (Fig. 1I; Movie 2), with motility inhibited in both the anterograde and retrograde directions (Fig. S1D). Imaging of EB1::GFP, a marker of growing plus ends of microtubules (Rogers et al., 2002), demonstrated that the axonal microtubules in the wing sensory neurons have a classical orientation, with plus ends extending in the anterograde direction (Fig. S1E; Table S2). Thus, both dynein and kinesin-1 are required for minus- and plus-end-directed transport of mitochondria in wing neurons, consistent with the mutually dependent relationship of these motors during trafficking of many organelles (Jolly and Gelfand, 2011; Martin et al., 1999; Sainath and Gallo, 2015).

The number of mitochondria undergoing transport in axons was also strongly reduced in both directions by an RNAi construct targeting Milton or a *UAS-Miro* overexpression construct that has a dominant-negative effect (Russo et al., 2009) (Fig. 1I; Fig. S1D; Movie 3). Disrupted minus-end-directed and plus-end-directed motion of mitochondria have previously been observed in other systems when Miro or Milton/TRAK function was inhibited (Russo et al., 2009; van Spronsen et al., 2013). Collectively, our observations indicate that mitochondrial motility can be characterised in detail in the wing nerve and that the transport machinery in this system is similar to that deployed in other neuronal cell types.

### Wing sensory neurons exhibit a boost and progressive decline in mitochondrial transport during adulthood

**Fig. 2.**
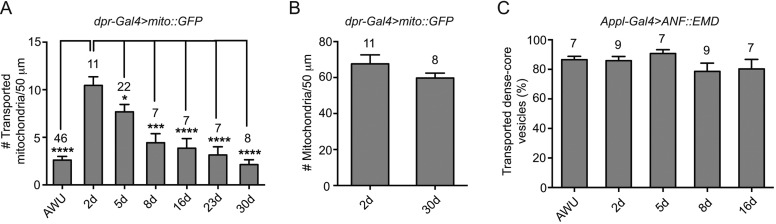
**Age-related changes in transport frequency of mitochondria but not DCVs in the wing nerve.** (A,B) Quantification of number of transported mitochondria (A) and total number of mitochondria (B) per 50 μm of axonal tract at different stages after eclosion. (C) Percentage of DCVs in 50 μm of the wing arch that are transported at different stages after eclosion. DCVs were fluorescently labelled with *UAS-ANF::EMD* under the control of *Appl-Gal4*. This pan-neuronal driver was used because the *dpr-Gal4* transgene is closely linked to a transgene that weakly expresses cytoplasmic GFP and thus hinders the visualisation of DCVs, which are much smaller than mitochondria. In control experiments, we confirmed that the age-related boost and decline in the proportion of transported mitochondria observed with *dpr-Gal4* was also seen with *Appl-Gal4* (Fig. S1J). As both *Appl-Gal4* and *UAS-ANF::EMD* are inserted on the X chromosome, imaging was performed only from female flies. d, day; AWU, after wing unfolding (within 20 min after wing unfolding, which typically occurs within 30–60 min after eclosion). The number of wings analysed is given above each bar; for each wing, a movie was captured for 3 min (A,B) or 2 min (C). Data are expressed as mean±s.e.m. *P<0.05; ***P<0.001; ****P<0.0001 (one-way ANOVA with Holm–Sidak's multiple comparison test).

We next performed a series of experiments investigating whether mitochondrial transport changes over time in adult wing nerve axons. We analysed mitochondrial motility in the wing arch of *dpr-Gal4 UAS-mito::GFP* animals at several points during the first 30 days of adulthood (Fig. 2A). In newly eclosed adults just after wing unfolding (AWU), few mitochondria underwent active transport (Fig. 2A; Movie 4). After 2 days, there was a large increase in the number of transported mitochondria (Fig. 2A; Movie 4). After the 2-day time point there was a progressive decline in the number of mitochondria transported (e.g. Movie 4), with a statistically significant reduction already evident at day 5 (Fig. 2A). This decline reflected a change in the proportion of mitochondria that was transported, as the total number of mitochondria in axons was not significantly different between 2 and 30 days after eclosion (Fig. 2B). The proportion of mitochondria that was transported at day 2 (16%) was less than we observed in our previous experiments at day 1 (30%), and this relative difference was confirmed by side-by-side comparison of transport at the two stages in an additional experimental series (Fig. S1F,F′). Thus, transport of these organelles peaks very early in adult life.

With the exception of a trend towards partially reduced retrograde run lengths, the motile properties of the transported subset of mitochondria did not change between 2 and 30 days after eclosion (Fig. S1G,H). Thus, the predominant effect on mitochondrial motility after 2 days of adulthood is a reduced likelihood of initiating directional transport. Interestingly, anterograde and retrograde run lengths and velocities of transported mitochondria were significantly lower at AWU than at later stages (Fig. S1G,H). At AWU, a sizeable fraction of mitochondria exhibited short-range oscillatory behaviour that was not observed at other stages (Movies 4 and 5). This was not due to a mixed polarity microtubule cytoskeleton because imaging of EB1::GFP revealed that, at this stage also, all growing plus ends extended in the anterograde direction (Table S2). We captured several occasions at AWU when oscillatory movements were converted into a bout of transport in the anterograde or retrograde direction (Fig. S1I; Movie 5), consistent with resolution of a tug-of-war between dynein and kinesin-1 motors bound to the organelle (Soppina et al., 2009). An increased tendency of the opposite motors to engage with the microtubule could conceivably explain reduced run lengths and velocities of transported mitochondria at AWU compared to later stages. Collectively, our data demonstrate a boost and subsequent decline in mitochondrial transport in wing neurons during early adulthood.

We next evaluated whether another cargo in wing neurons shows the same age-associated changes in transport by expressing rat prepro-atrial natriuretic factor peptide (ANF) tagged with Emerald (EMD) (Rao et al., 2001), a fluorescent marker of dense-core vesicles (DCVs), in the wing nerve. Consistent with observations in other neurons (Barkus et al., 2008; Lo et al., 2011), initial studies at 2 days after eclosion revealed long-distance axonal transport of DCVs in both directions, including a fraction of vesicles that exhibited instantaneous directional reversals (Fig. S2A,B; Movie 6). Interestingly, monitoring DCV transport between AWU and 16 days after eclosion revealed no change in the proportion of vesicles that underwent active transport, with 80–90% motile at all stages examined (Fig. 2C). We also did not observe a decline in the velocities and run lengths of transported DCVs during ageing (Fig. S2C). Thus, the changes in motility of mitochondria observed in wing neurons of ageing flies do not reflect generalised changes in axonal cargo transport.

### Inhibiting mitochondrial motility in adult stages accelerates the appearance of focal protein accumulations in ageing neurons

**Fig. 3.**
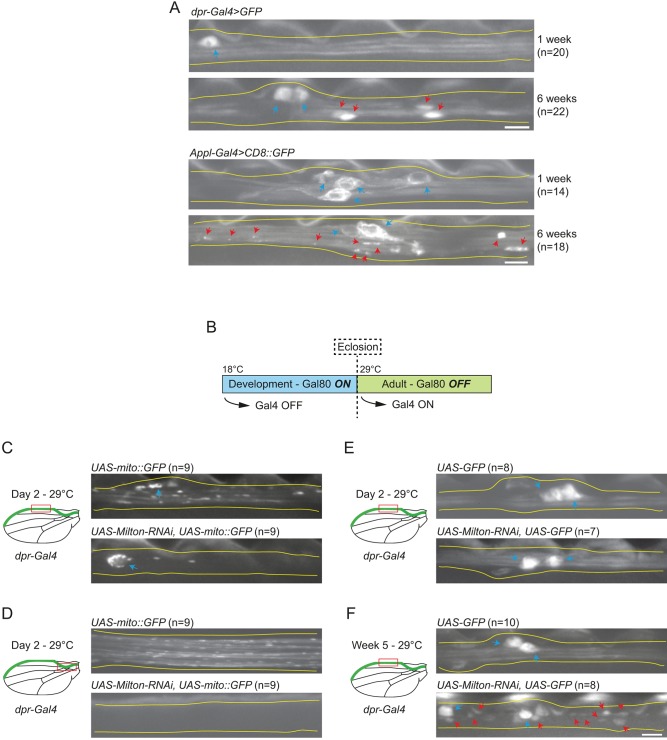
**Inhibiting mitochondrial transport in adult wing neurons accelerates age-related focal accumulation of proteins.** (A) Representative images of wing neurons exhibiting age-dependent focal accumulations of cytoplasmic GFP or CD8::GFP at 6 weeks after eclosion. Focal accumulations are not seen 1 week after eclosion. In A and C–F: red arrows, focal accumulations of GFP proteins; blue arrows, cell bodies; yellow lines, outline of the wing nerve; *n*, number of wings imaged. (B) Schematic of method used to interfere with mitochondrial transport specifically in adult stages. (C,D) Representative images of wing nerve axons after shifting *dpr-Gal4 tubulin-Gal80^ts^ UAS-mito::GFP* flies to 29°C for 2 days after eclosion in the absence or presence of the *UAS-Milton*-*RNAi* transgene. Images are from the margin (C) or arch (D) regions (red boxes in cartoons). The RNAi condition leads to very few labelled mitochondria in axons in either region. (E,F) Representative images of wing nerve axons after shifting *dpr-Gal4 tubulin-Gal80^ts^ UAS-GFP* flies to 29°C for 2 days (E) or 5 weeks (F) after eclosion in the absence or presence of the *UAS-Milton*-*RNAi* transgene. Images are from the margin region (red box in cartoons). The RNAi condition leads to large focal accumulations of GFP in the axons by 5 weeks. Scale bars: 5 μm.

Several studies have reported an age-associated decline in protein homeostasis in adult neurons, as evidenced by progressive focal accumulations of soluble and membrane proteins (Cohen and Dillin, 2008; David et al., 2010; Goedert et al., 1988; Mitchell et al., 2015; Powers et al., 2009; Spillantini et al., 1997; Zou et al., 2015). This decline also appears to occur in the wing nerve as ageing axons exhibited focal accumulations of cytoplasmic GFP and GFP fused to a transmembrane domain (from CD8; CD8::GFP), which were first visible during week 5 or 6 after eclosion (Fig. 3A). The decline in mitochondrial transport in the wing nerve precedes the appearance of focal protein accumulations. This raises the possibility that the progressive reduction in mitochondrial motility during adult life contributes, at least in part, to a decline in protein homeostasis.

To explore this possibility, we first asked whether transport of mitochondria in adult neurons is required to maintain protein homeostasis. Alternatively, the distribution of these organelles during development could be sufficient to sustain this process in later life. To address this issue, we induced the expression of the *UAS-Milton*-*RNAi* construct specifically in *dpr^+^* neurons in the adult. This was achieved by combining *dpr-Gal4* with a temperature-sensitive version of the Gal4 repressor, Gal80^ts^ (Pfeiffer et al., 2010; Suster et al., 2004) (Fig. 3B). In control experiments, we confirmed that *dpr-Gal4* was inactive at the temperature permissive for Gal80^ts^ function (18°C; Fig. S3A). We subsequently performed experiments in which flies were shifted to the restrictive temperature (29°C) shortly after eclosion, thus initiating expression of the *Milton* RNAi under the control of *dpr-Gal4*. The *UAS*-*mito::GFP* transgene was also included in the genotype so that mitochondria could be visualised.

Expression of the *UAS-Milton*-*RNAi* construct for 2 days after eclosion resulted in very few labelled mitochondria in the proximal regions of the axon and none in more distal regions (Fig. 3C,D). In contrast, GFP-labelled mitochondria were distributed throughout axons following identical treatment of flies containing *Gal80^ts^*, *dpr-Gal4* and *UAS*-*mito::GFP* but no RNAi construct (Fig. 3C,D). Axonal depletion of labelled mitochondria was sustained when the flies with the *Milton* RNAi construct were incubated at the restrictive temperature for the first 2 weeks of adult life, whereas control flies treated in the same manner had a wild-type axonal distribution of these organelles (Fig. S3B). Adult-specific inhibition of Milton typically resulted in an increase in the mito::GFP signal in the neuronal cell body compared to the control situation (Fig. S3B). These observations demonstrate that strongly inhibiting transport of mitochondria in adult life is sufficient to disrupt trafficking of these organelles into axons.

We next used the Gal4-Gal80^ts^ system to inhibit mitochondrial transport in adult *dpr^+^* neurons with *UAS-Milton*-*RNAi* and simultaneously express cytoplasmic GFP, which acted as a marker of protein homeostasis. Flies were again shifted to 29°C shortly after eclosion. When no RNAi construct was present, GFP was distributed uniformly within axons up to 5 weeks after the shift to the restrictive temperature (Fig. 3E,F). In contrast, in all wings examined, RNAi of *Milton* in adult neurons resulted in many large axonal accumulations of GFP by 5 weeks after eclosion (Fig. 3F; Fig. S3C). The appearance of these accumulations was progressive and not due to non-specific deployment of the RNAi machinery (Fig. S3D,E). We conclude that adult-specific downregulation of mitochondrial transport in wing neurons accelerates the appearance of focal protein accumulations.

### Reducing Lissencephaly-1 levels increases mitochondrial motility in adult wing neurons

**Fig. 4.**
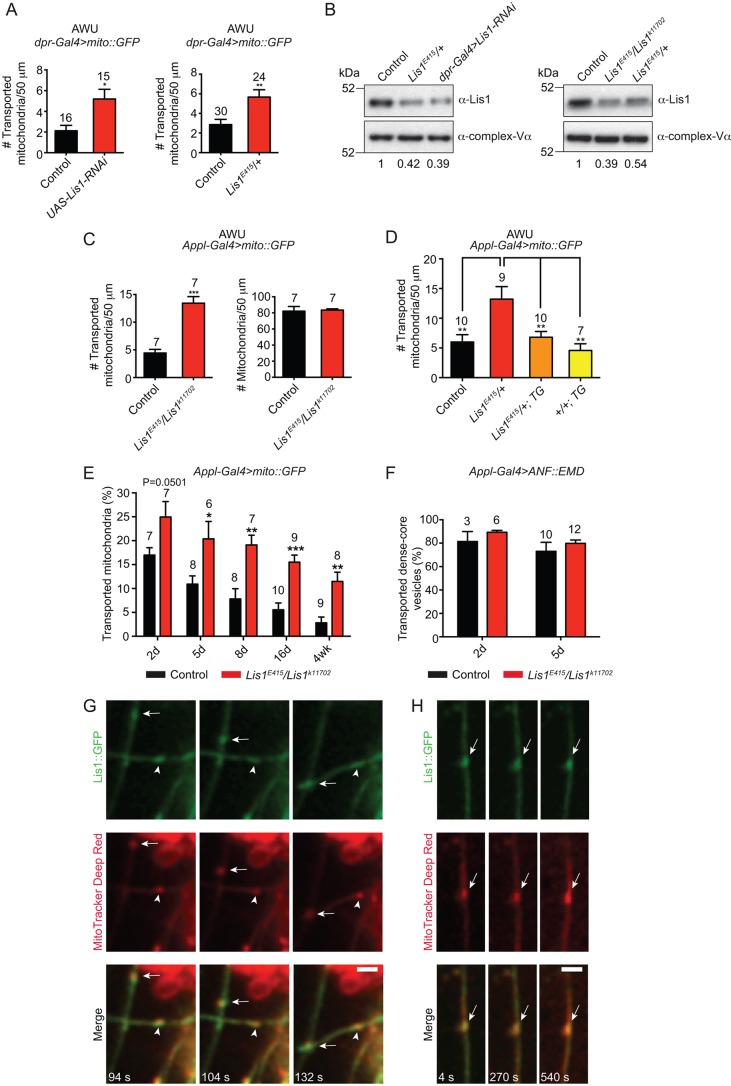
**Lis1 can negatively regulate mitochondrial transport in wing neurons and associate with mitochondria in S2R+ cells.** (A) Quantification of the number of transported mitochondria in axons of *dpr^+^* neurons at AWU. Control genotype is *dpr-Gal4 UAS-mito::GFP* only. (B) Immunoblots from wing extracts showing Lis1 levels. Loading control, mitochondrial complex-Vα; control genotype, Oregon-R. Numbers indicate the ratio of Lis1 to complex-Vα signals for each sample. The values obtained are similar to those obtained by quantitative western blotting of embryo extracts from these genotypes (Dix et al., 2013). (C) Number of mitochondria that are transported and total number of mitochondria per 50 μm at AWU in axons of *Appl^+^* neurons in *Lis1^E415^/Lis1^k11702^* mutants. *Appl-Gal4* was used in the *Lis1* trans-heterozygous background as the presence of *dpr-Gal4* and *Lis1* on the same chromosome complicates genetic crosses. Mean number of transported mitochondria in the control is higher with *Appl-Gal4* than with *dpr-Gal4* (A) due to expression of the driver in a broader set of sensory neurons. (D) A ubiquitously expressed Lis1 transgene (TG) driven by the α-tubulin promoter (Dix et al., 2013) suppresses the increase in mitochondrial motility observed at AWU in *Lis1^E415^/+* axons. (E) Percentage of transported mitochondria in axons of *Appl^+^* neurons at different stages during the first 4 weeks after eclosion in *Lis1^E415^/Lis1^k11702^* mutants. In C–E, the control genotype is *Appl-Gal4 UAS-mito::GFP* only*.* (F) Percentage of DCVs that are transported at 2 and 5 days after eclosion in *Lis1^E415^/Lis1^k11702^* mutants. Control genotype, *Appl-Gal4 UAS-ANF::EMD* only*.* The dataset at 5 days is produced from the combination of two experiments, both of which did not show a significant difference between the genotypes. (G,H) Stills from Movies 8 and 9 showing that Lis1::GFP associates with transported (G) or stationary (H) mitochondria in processes of S2R+ cells. Arrowhead, retrogradely moving mitochondrion; arrow, anterogradely moving mitochondrion. Retrograde and anterograde movements in processes are minus-end- and plus-end-directed, respectively (Ling et al., 2004). Ten cells were imaged from four technical replicates. d, day; wk, week. In A and C–F, the number of wings analysed is given above each bar with data expressed as mean±s.e.m.; for each wing, a movie was captured for 3 min (A,C–E) or 2 min (F). *P<0.05; **P<0.01; ***P<0.001 [two-tailed Student's *t*-test compared to control values at the same stage (A,C,E,F) and a one-way ANOVA with Holm–Sidak's multiple comparison test (D)]. Scale bars: 3 μm.

In order to further explore the potential link between mitochondrial transport and protein homeostasis in adult neurons, we sought to boost mitochondrial transport in wing neurons and assess the phenotypic consequences. In a concurrent project (unpublished observations), we performed a candidate-based RNAi screen for factors that regulate mitochondrial motility in wing neurons. As part of the screen, we examined flies at AWU that expressed an RNAi construct targeting the dynein co-factor Lissencephaly-1 (Lis1) together with mito::GFP in *dpr^+^* neurons. An approximately twofold increase in the number of transported mitochondria was observed with *Lis1* RNAi compared to the control (Fig. 4A). Immunoblotting of whole wings [in which the sensory neurons make up a large fraction of cells (Johnson and Milner, 1987; Kiger et al., 2007)] confirmed reduction of Lis1 protein levels in the RNAi experiment (Fig. 4B).

Lis1 is so-named because haploinsufficiency for the human gene causes lissencephaly, a developmental brain disorder characterised by defective neuronal migration. The protein associates directly with the motor domain of dynein (Huang et al., 2012; McKenney et al., 2010; Sasaki et al., 2000; Tai et al., 2002), with several *in vitro* studies showing that Lis1 increases the affinity of the dynein complex for microtubules without binding microtubules itself (Huang et al., 2012; McKenney et al., 2010; Toropova et al., 2014). Lis1 can also promote the association of dynein and its accessory complex dynactin with at least some cargoes (Dix et al., 2013).

Increased initiation of mitochondrial motility when Lis1 levels were reduced was surprising because several previous studies have reported that lowering the concentration of this protein inhibits transport of cargoes by dynein (see Discussion). We therefore used classical mutant alleles to confirm that inhibiting Lis1 function boosts mitochondrial transport. As is the case in the mouse (Hirotsune et al., 1998), null mutations in *Lis1* in *Drosophila* result in lethality during developmental stages (Liu et al., 2000). We therefore examined genotypes in which Lis1 protein levels are partially reduced. Lowering Lis1 levels with one copy of the *E415* allele of *Lis1* (which contains an insertion of a P-element transposon in the gene locus) significantly increased the number of transported mitochondria in wing axons at AWU (Fig. 4A,B). Combining the *E415* allele with another P-element allele of *Lis1* (*k11702*), which further decreases Lis1 protein levels (Fig. 4B; Dix et al., 2013), gave a stronger increase in the number of transported mitochondria at this stage (Fig. 4C). We also observed a significant increase in the number of motile mitochondria at AWU using one copy of another *Lis1* P-element allele (*k13209*; Fig. S4A). The increase in the number of motile mitochondria in *Lis1* mutant wing neurons could be suppressed by a ubiquitously expressed Lis1 transgene (Fig. 4D), confirming that the reduced Lis1 levels are responsible for the phenotype. In each of the *Lis1* genotypes, an increase in the total number of mitochondria was not evident compared to the controls, and this was confirmed for *Lis1^E415^/Lis1^k11702^* flies by quantitative analysis (Fig. 4C). We conclude that lowering Lis1 levels increases the proportion of motile mitochondria at AWU.

To determine whether the effect of Lis1 on mitochondrial motility is restricted to this early stage, we examined mitochondrial transport in wing neurons of *Lis1^E415^/Lis1^k11702^* animals at several time points during the first 4 weeks following eclosion. There was an age-related decline in the proportion of mitochondria undergoing active transport in *Lis1^E415^/Lis1^k11702^* neurons (Fig. 4E). However, at each stage examined there was a 1.5- to 4-fold increase in the frequency of mitochondrial transport events compared to the same stage in the control (Fig. 4E; Movie 7). For example, the proportion of motile mitochondria in the *Lis1* mutant neurons at 4 weeks was similar to that observed at day 5 for the control (Fig. 4E). Increases in mitochondrial transport occurred in both the retrograde and anterograde direction in *Lis1^E415^/Lis1^k11702^* axons (Fig. S4B), and were not associated with an increase in the total number of mitochondria (Fig. S4C). We conclude that Lis1 suppresses mitochondrial transport at several stages of adult life. To further explore the effects of reducing Lis1 levels on cargo transport, we evaluated motility of DCVs at two different time points in *Lis1^E415^/Lis1^k11702^* wing axons. There was no significant difference in the proportion of motile DCVs in the mutant compared to the wild-type (Fig. 4F). Thus, reducing *Lis1* levels does not cause a generalised increase in cargo transport.

At most stages examined, run lengths and velocities of mitochondria in *Lis1^E415^/Lis1^k11702^* wing axons were similar to those in the wild-type condition (Fig. S4D,E). This indicates that Lis1 levels predominantly affect the proportion of mitochondria that undergo active transport rather than motile properties following transport initiation. We did, however, observe a modest increase in anterograde velocities of transported mitochondria in *Lis1* mutants at all stages (Fig. S4D). Increased anterograde velocity has also been reported for the motile subset of lysosomes and/or late endosomes in cultured mammalian cells (Yi et al., 2011), and for mRNAs in *Drosophila* embryos (Dix et al., 2013), when Lis1 was inhibited. The mechanistic basis for increased anterograde velocity is unclear but could conceivably be due to reduced drag on kinesin-1-driven plus end movement when the affinity of dynein for microtubules is reduced. Overall, our data suggest that Lis1 plays a role in restraining microtubule-based motion of mitochondria in the wing nerve.

As Lis1 can bind the dynein motor, it is conceivable that it regulates the motility of mitochondria by associating with these organelles. In many cell types, the high cytoplasmic concentration of motor complex components prevents their detection on cargoes. We therefore used cultured *Drosophila* S2R+ cells to test for an association of Lis1 with mitochondria. These cells can be induced to form very thin processes, which are well-suited to the visualisation of cargo–motor complexes by light microscopy (Lu et al., 2013). S2R+ cells were transfected with a plasmid encoding Lis1::GFP, and the cell processes were then induced (Fig. S4F). Subsequently, the cells were incubated with the vital mitochondrial dye MitoTracker Deep Red. We often observed enrichment of Lis1::GFP on mitochondria in cell processes (Fig. 4G,H; Movies 8 and 9). This enrichment was observed on mitochondria that were stationary, or undergoing minus-end- or plus-end-directed transport (Fig. 4G,H; Movies 8 and 9). In control experiments, we confirmed that GFP alone was not enriched on mitochondria in S2R+ cell processes (Fig. S4G). We conclude that Lis1 has the capacity to associate with mitochondria, and therefore might directly regulate their transport.

### Reducing Lis1 levels retards the formation of age-dependent focal accumulations of proteins

**Fig. 5.**
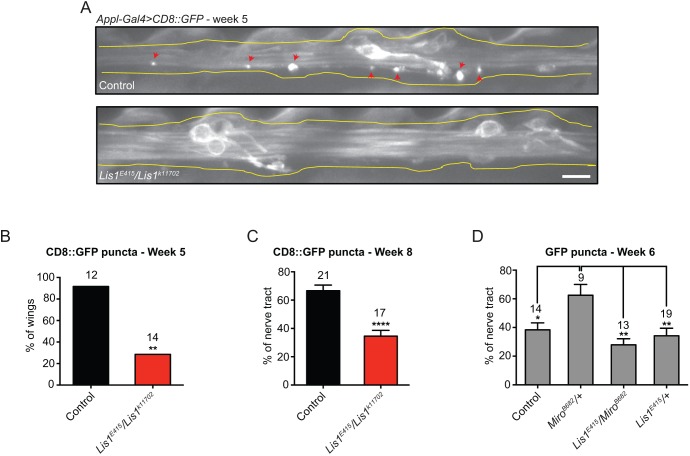
**Reducing Lis1 levels retards focal accumulation of proteins in axons during ageing.** (A) Representative images of CD8::GFP in control or *Lis1^E415^/Lis1^k11702^* axons of *Appl^+^* neurons. Control genotype, *Appl-Gal4 UAS-CD8::GFP* only*.* Red arrows, focal accumulations of CD8::GFP; yellow lines, approximate outline of the wing nerve. Scale bar: 5 μm. (B) Proportion of wings containing CD8::GFP focal accumulations at 5 weeks. (C) Extent of wing nerve affected by CD8::GFP focal accumulations at 8 weeks. Control genotype is *Appl-Gal4 UAS-CD8::GFP* only*.* (D) Extent of wing nerve of *dpr^+^* neurons affected by focal accumulations of cytoplasmic GFP at 6 weeks. Control genotype, *dpr-Gal4 UAS-GFP* only. In B–D, the number of wings analysed is given above each bar, with data expressed as mean±s.e.m. Values in C and D were calculated by determining how many segments of the wing nerve contained at least one accumulation of the GFP protein (see Materials and Methods). *P<0.05; **P<0.01; ****P<0.0001 [Fisher's exact test (B), two-tailed Student's *t*-test (C) or one-way ANOVA with Holm–Sidak's multiple comparison (D)].

We next asked whether the increase in axonal transport of mitochondria in *Lis1* mutants is accompanied by the delayed appearance of focal protein accumulations during ageing. We first expressed CD8::GFP in *Lis1^E415^/Lis1^k11702^* and wild-type animals with *Appl-Gal4* and examined wing nerves. In the wild-type background, ∼90% of wings contained a small number of CD8::GFP accumulations by 5 weeks after eclosion (Fig. 5A,B). These puncta were typically localised to a small region of the axonal tract. In contrast, only 30% of *Lis1^E415^/Lis1^k11702^* wings contained visible CD8::GFP accumulations at this stage (Fig. 5A,B). By 8 weeks of adult life, these puncta were evident in almost all wing axons of both *Lis1^E415^/Lis1^k11702^* and control animals. However, the fraction of the axonal length that was affected by CD8::GFP accumulation was substantially lower in the *Lis1* mutant neurons compared to wild-type (Fig. 5C). Collectively, these data indicate that reducing Lis1 levels protects against the appearance of age-related protein accumulations in adult neurons.

We next performed genetic interaction experiments to explore the link between elevated mitochondrial transport events in *Lis1* mutants and the observed effect on focal protein accumulations. Heterozygosity for a null allele of *Miro* partially reduced the frequency of mitochondrial transport events in axons of *dpr^+^* wing neurons (Fig. S4H), consistent with previous observations in axons of larval motor neurons (Russo et al., 2009). The decrease in mitochondrial transport in wing neurons was accompanied by increased focal accumulations of cytoplasmic GFP during ageing (Fig. 5D). This observation provides further evidence of a link between mitochondrial motility and efficient protein homeostasis. Strikingly, the increase in focal protein accumulations in the *Miro* heterozygotes was completely suppressed by heterozygosity for the *Lis1^E415^* allele (Fig. 5D). Thus, reducing Lis1 levels can attenuate protein homeostasis defects caused specifically by targeting the mitochondrial transport machinery. This observation is consistent with *Lis1* mutations suppressing the onset of age-related axonal phenotypes, at least in part, because of augmented mitochondrial transport.

## DISCUSSION

### Utility of the *Drosophila* wing for axonal transport studies in adult neurons

Our mounting and imaging procedures allow axonal transport to be visualised in detail in the wing nerve. Compared to other systems for intravital imaging of cargo transport in adult neurons, the wing nerve has significant advantages. First, longitudinal studies are much less time-consuming than in vertebrate model organisms due to the short lifespan of *Drosophila*. Second, sample preparation is simple, rapid and non-invasive. Third, the sophisticated genetic tools available in *Drosophila* facilitate manipulation of the transport process. The wing nerve system does have some drawbacks, not least the inability to perform whole-mount immunostaining and large-scale biochemistry. These issues are, however, shared with several other systems for intravital imaging of cargo transport in adult neurons.

### Lis1 can negatively regulate mitochondrial transport in adult wing neurons

We discovered that *Lis1* mutants with reduced protein levels have a substantial boost in the proportion of mitochondria undergoing active transport in the wing nerve. This effect appears to reflect a cell autonomous function of Lis1 because the same phenotype was observed when the level of the protein was lowered specifically in *dpr^+^* neurons using a *UAS-RNAi* construct. Our data therefore indicate that Lis1 functions in wing sensory neurons to determine the fraction of motile and static mitochondria.

We observed colocalisation of Lis1 with both static and motile mitochondria in processes of S2R+ cells, which are well suited to imaging of cargo–motor complexes. The association of Lis1 with motile mitochondria challenges the model that the protein must dissociate from microtubule-associated cargoes before transport begins (Egan et al., 2012; Yamada et al., 2013). The ability of Lis1 to associate with mitochondria, together with its well-characterised biochemical interaction with dynein, indicates that it could regulate mitochondrial motility as a component of the transport machinery. Interestingly, reducing Lis1 levels leads to increased active transport of mitochondria in the plus end direction as well as the minus end direction in the wing nerve. This observation provides further evidence of tight coupling between the activities of dynein and kinesin-1 on cargoes (Jolly and Gelfand, 2011).

It has previously been observed in other cell types and developmental stages that reducing Lis1 function inhibits dynein-dependent translocation of nuclei (Tsai et al., 2005; Xiang et al., 1995), lysosomes (Klinman and Holzbaur, 2015; Moughamian et al., 2013; Pandey and Smith, 2011), mRNA (Dix et al., 2013) and endosomes (Egan et al., 2012; Lenz et al., 2006; Yi et al., 2011). Indeed, the same *Lis1* mutant genotypes that boost mitochondrial transport in the *Drosophila* wing (*Lis1^E415^*/+ and *Lis1^E415^/Lis1^k11702^*) were previously found to reduce mRNA transport in the embryo (Dix et al., 2013). Of particular relevance for our current study, it has been reported that RNAi-based knockdown of Lis1 arrests axonal mitochondrial transport in cultured rat-embryo-derived hippocampal neurons (Shao et al., 2013).

The seemingly contradictory effects of Lis1 inhibition on mitochondrial transport in adult wing neurons could be because the protein is an activator of dynein-based transport of specific cargoes in some cell types or developmental stages, and an inhibitor of transport in others, for instance by co-operating with different co-factors. Alternatively, the well-characterised ability of Lis1 to increase the affinity of dynein for microtubules might have different consequences in different contexts when Lis1 function is partially inhibited. Several cargoes might be unable to engage microtubules in the first place, or have an increased rate of detachment following transport initiation, when the affinity of dynein for microtubules is reduced by lowering Lis1 levels. This would result in impaired transport. In contrast, cargoes such as mitochondria in the wing nerve could have sufficient affinity for microtubules for effective engagement of the cargo–motor assembly with the track when Lis1 is partially inhibited. Subsequently, the reduced binding strength of dynein to the track could increase the likelihood of transport initiation compared to the wild-type situation. It is not clear why mitochondria in the system we studied would have a relatively high affinity for microtubules. However, this could conceivably be due to a high copy number of motors or the use of additional microtubule-tethering factors (Chen and Sheng, 2013; Kang et al., 2008). Whatever the mechanism, the implication for wild-type cells is that controlling Lis1 activity is an effective strategy for regulating the balance between loading of different cargoes on microtubules and the initiation of their transport. Additional studies will be required to test this speculative hypothesis.

### Insights into dynamics of mitochondrial transport in adult wing neurons

Our analysis of axonal transport over time reveals a boost followed by a decline in the proportion of motile mitochondria in the wing nerve in the first week of adult life. The boost occurs during the first day after the extension of the fly wing at the end of the ecdysis sequence, which marks the conclusion of fly development and the beginning of adulthood (Kimura et al., 2004; Peabody et al., 2008). The subsequent decline in transport is already evident by 2 days after eclosion and continues through all subsequent time points analysed. These changes occur equally in the anterograde and retrograde direction, and are not accompanied by an altered density of mitochondria in the axon.

Several investigations have provided evidence that the transport of at least some cargoes declines during the lifespan of rodent models (McQuarrie et al., 1989; Uchida et al., 2001; Viancour and Kreiter, 1993). A decline in the proportion of motile mitochondria has recently been observed in the peripheral nerve and central nervous system of adult mice (Milde et al., 2015), with a reduction in motility already evident between 3 and 6 months after birth. Thus, an age-related reduction in mitochondrial transport appears to be conserved in adult neurons of different species. Milde et al. did not examine axonal transport in very young animals (before 6 weeks of age), so it is not known whether the boost in transport we observe in the wing nerve of young flies is also conserved. The authors did observe a decline in the transport of Golgi-derived vesicles at later time points, raising the possibility of generalised changes in axonal transport during ageing. Our finding that the frequency of DCV transport events does not change over the first weeks of adult life indicates that, at least in *Drosophila*, there is a process that affects transport of a subset of cargoes.

The molecular basis of age-related changes in the proportion of mitochondria transported in the mouse nervous system and the *Drosophila* wing nerve is not known. We could not detect changes in Lis1 levels in wings as flies age (Fig. S4I), suggesting that altered concentrations of this protein are unlikely to be a key factor in the age-related changes in mitochondrial dynamics. We cannot, however, rule out that post-translational regulation of Lis1 plays a role in the age-related changes in motility. Future studies will exploit the advantages of the wing nerve system to investigate how mitochondrial transport is regulated over time, and if and how this mechanism impinges on Lis1 function.

### Evidence for links between mitochondrial motility and protein homeostasis in ageing neurons

The decline in mitochondrial motility in wing nerves precedes the onset of focal accumulations of cytoplasmic and membrane proteins that were observed during ageing. These observations raised the possibility of a contribution of reduced mitochondrial transport in ageing neurons to a decline in protein homeostasis. Consistent with this notion, strongly disrupting mitochondrial transport specifically in adult stages with the Gal80^ts^ system greatly accelerates the appearance of focal protein accumulations in axons. Elevated protein accumulation is also evident when mitochondrial transport is partially inhibited in *Miro* heterozygotes. Furthermore, we show that the sustained increase in mitochondrial transport in *Lis1* mutant wing axons is accompanied by a substantial delay in the appearance of focal protein accumulations. Reducing Lis1 levels also suppresses protein homeostasis defects caused by specifically inhibiting the mitochondrial transport machinery. We cannot rule out functions of Lis1 other than in mitochondrial transport influencing the delay in protein accumulations in *Lis1* mutants. However, the most parsimonious interpretation of our data set is that increased mitochondrial transport in the mutants contributes, at least in part, to this phenotype.

Collectively, our data provide novel evidence of a link between axonal transport of mitochondria and protein homeostasis. It has been previously shown that an approximately twofold increase in mitochondrial motility that occurs in mice lacking the microtubule-tethering factor syntaphilin does not affect the onset of ALS symptoms caused by an aggregrate-prone SOD-1 mutant protein (Zhu and Sheng, 2011). However, the authors acknowledged that the short lifespan resulting from mutant SOD-1 expression could mask potential long-term benefits of increased mitochondrial motility. Based on our data, we speculate that elevating axonal transport of these organelles can increase the healthy lifespan of neurons in some contexts.

In *Lis1* mutants, there is a balanced increase in retrograde and anterograde transport, and no change in the density of mitochondria in axons. This would suggest that any protective effect of increased mitochondrial transport in *Lis1* mutants is not associated with an increase in net supply of mitochondria to the axon. Instead, the increased motility of these organelles within the axon is likely to be the important factor. The importance of mitochondrial movement per se is also supported by the increase in age-related focal protein accumulations in *Miro* heterozygotes, in which transport, but not density, of mitochondria within the axon is partially reduced (Fig. S4H). Several possibilities exist for how mitochondrial movements within the axon could have a protective function (Avery et al., 2012; Court and Coleman, 2012; Schwarz, 2013; Sheng and Cai, 2012). These include (1) exposing a broader cytoplasmic area to the Ca^2+^ buffering and ATP supply functions of the organelles, (2) promoting encounters between mitochondria that facilitate exchanges of material and (3) recycling of damaged mitochondria back to the cell body for mitophagy. Distinguishing between these and other possible roles of axonal mitochondrial transport will be the goal of future studies. It will be also desirable to test whether partially reducing Lis1 activity in adult mammalian neurons can increase their healthy lifespan, including in neurodegenerative disease models.

## MATERIALS AND METHODS

### *Drosophila* strains and husbandry

Strains containing the following transgenes or mutant alleles were obtained from the Bloomington *Drosophila* Stock Center (Indiana University, IN): *dpr-Gal4* (also containing a closely linked *UAS-GFP* transgene) (BL#25083), *UAS-mito::GFP* (BL#8442); *UAS-mito::GFP* (BL#8443); *UAS-GCaMP6f* (BL#42747); *Appl-Gal4* (BL#32040); *UAS-preproANF::EMD* (BL#7001); *UAS-Miro* (BL#51646); *tubP-Gal80^ts^* (BL#7016); *Miro^B682^* (BL#52003); *Lis1^k13209^* (BL#11072); *UAS-Luciferase-RNAi* (BL#31603); *UAS-Lis1-RNAi* (BL#28663); *UAS-Milton-RNAi* (BL#44477); *UAS-Khc-RNAi* (BL#35770); and *UAS-Dhc-RNAi* (BL#36698). The RNAi lines were generated by the Transgenic RNAi Project (TRiP) at Harvard Medical School, MA. *Lis1^E415^* and *Lis1^k11702^* alleles are described elsewhere (Dix et al., 2013; Swan et al., 1999). The *α-tubulin–Lis1* transgene is described in Dix et al. (2013). *UAS-EB1::GFP* (Fabrowski et al., 2013) and *UAS-CD8::GFP* were gifts from Damian Brunner (University of Zurich, Switzerland) and Hannah Salter (MRC-LMB, Cambridge, UK), respectively. Control animals were generated by crossing a *dpr-Gal4 UAS-mito::GFP* stock to Oregon-R (wild-type) flies or by crossing *Appl-Gal4* flies to *UAS-mito::GFP* flies.

Animals were collected each day after eclosion and transferred into vials containing fresh food once or twice per week following anaesthetisation. Flies from the same experimental series were transferred to new vials containing the same batch of food on the same day. Flies were cultured on ‘Iberian’ food [70 mg/ml yeast (*Saccharomyces cerevisiae*, Type II, Sigma-Aldrich), 55 mg/ml glucose (Formedium), 7.7 mg/ml agar (*Drosophila* Agar Type II, Dutscher Scientific), 35 mg/ml organic plain white flour (BigBarn CIC, UK), 1.2 mg/ml Tegosept (Dutscher Scientific), 0.4% propionic acid (Sigma-Aldrich)]. For standard culture, flies were maintained at 25°C and 50% humidity with a 12-h-light–12-h-dark cycle. For *Gal80^ts^* experiments, flies were reared at 18°C throughout development and shifted to 29°C typically within 1 h of eclosion.

### Live imaging and quantification of axonal transport

Flies were anaesthetised with CO_2_ and enclosed in a custom-built chamber formed of two No. 0 coverglasses (22×64 mm; Scientific Laboratory Supplies). Four layers of tape (three layers of masking tape with a layer of double-sided tape on top) were placed along each of the two short edges of the lower coverglass. The coverglass was covered with a thin coat of 10S halocarbon oil (VWR) and the fly immobilised ventral side up by placing the head on one piece of double-sided tape. The wings were covered with additional 10S halocarbon oil. The upper coverglass was gently placed on the lower coverglass to prevent movements of the animal during imaging. Flies survived the mounting and imaging procedure and could be returned to the food. However, as the wings were covered in oil they often became attached to the body the animal. Thus, imaging from the same flies at multiple time points was not performed.

Imaging was performed at a constant temperature of 21–22°C with a spinning disk imaging system (UltraVIEW ERS; PerkinElmer) using an IX71 inverted microscope (Olympus) equipped with a CCD camera (Orca ER, Hamamatsu) and a 60×/1.4 NA PlanApo oil-immersion objective. Image series were only captured from wings that did not show signs of damage. A single focal plane was imaged with an acquisition rate of 1 frame/s (DCVs and EB1::GFP) or 0.5 frame/s (mitochondria). Exposure times were 0.5 s (DCVs), 0.65 s (EB1) or 0.3 s (mitochondria). Extending the period of imaging to 4 h did not interfere with mitochondrial motility or morphology, suggesting that the imaging procedure does not have a detrimental effect on transport.

Quantification of movements was performed in ImageJ. A 50-μm region of the axonal tract of the wing arch region was selected and straightened with the Straighten plugin (Eva Kocsis, NIH, MD). Where needed, straightened image series were stabilised with the StackReg plugin (Philippe Thévenaz, EPFL, Switzerland) or the Image Stabilizer plugin (Kang Li, Carnegie Mellon University, PA). Operationally, transported particles were defined as those containing at least one continuous bout of net motion of at least 2 μm (a ‘run’). By this definition, the oscillatory movements of mitochondria that were prevalent at AWU were not considered as transport. Transported mitochondria were manually tracked with MTrackJ (Meijering et al., 2012) by recording the start and the end of each run. Tracking was stopped and the run terminated if the transported particle moved out of the focal plane. Run length and velocity values were exported into Excel (Microsoft) for analysis, followed by data plotting using Prism (GraphPad). Kymographs were plotted with the Kymograph Creator plugin of ImageJ. Total mitochondria were typically counted from a single frame with the Cell Counter plugin of ImageJ (Kurt de Vos, University of Sheffield, UK).

### Imaging of focal protein accumulations in wing neurons

Flies overexpressing GFP or CD8::GFP were mounted in the imaging chamber and imaged as described above. For GFP expression, we used the *UAS-GFP* transgene present in the *dpr-Gal4* stock. *z*-stacks of neurons were acquired from several focal planes. The ‘Smooth’ filter of ImageJ (which replaces each pixel with the average of its 3×3 neighborhood pixels) was applied to *z*-projections of stacks in Fig. 3 and Fig. S3 before assembling the figures. The different genotypes within a single experiment were imaged with the same laser power and camera gain, although occasionally exposure time varied slightly to account for fluctuations in fluorescence between different mounted wings.

The following method was used to score the percentage of wing nerve affected by focal protein accumulations (Fig. 5C,D). The nerve tract was divided into four portions of roughly equal length: (1) the wing arch; (2) the L1–costal-vein intersection to the mid-margin (measured by counting nine or ten dorsal chemosensory bristles); (3) the mid-margin to the L1–L2 vein intersection; (4) the L1–L2 intersection to the tip of the wing (i.e. where the cell body of the last neuron is observed). Each of the four portions was then further divided into two equal sub-regions. The number of sub-regions containing one or more focal accumulations was scored, with each assigned a value of 12.5%.

### Preparation of wing extracts and immunoblotting

Protein extracts from wings were prepared following a protocol adapted from Fang et al. (2012). Wings were collected by cutting at the wing root close to the thorax of the animal with fine spring scissors. Typically, 60 wings were collected in a Dounce homogeniser on ice and homogenised in 300 µl of lysis buffer containing 50 mM Tris-HCl pH 7.4, 150 mM NaCl, 5 mM EDTA, 1× PhosSTOP phosphatase inhibitor (Roche) and 1× Complete Protease Inhibitor (Roche). After addition of Triton X-100 to a final concentration of 1%, the samples were kept on ice for 10 min and tubes flicked every 2 min to aid solubilisation. Lysates were spun at 16,100 ***g*** for 30 min at 4°C. 50 mM DTT was added to the supernatant, mixed with LDS sample buffer (Novex-Life Technologies) and boiled for 10 min at 90°C. Following gel electrophoresis and protein transfer, membranes were incubated successively with rabbit anti-Lis1 (Dix et al., 2013) (diluted 1:1000) or mouse anti-Complex-Vα primary (MitoSciences-Life Technologies, clone 15H4C4) (diluted 1:5000) antibodies, horseradish peroxidase (HRP)-conjugated secondary antibodies and Immobilon chemiluminescent substrate (Merck-Millipore). The specificity of the Lis1 antibody has been validated using mutant extracts (Dix et al., 2013). The anti-ComplexVα antibody recognises *Drosophila* mitochondria in immunohistochemistry and results in a single band of the expected molecular mass in immunoblots. Typically, between 3% and 8% of total lysate sample was loaded per gel lane. Immunoblot signals were calculated with the gel analysis function of ImageJ.

### Imaging *Drosophila* S2R+ cells

Lis1::GFP or GFP alone was expressed from a pCASPER-based plasmid containing the *α-tubulin* promoter, the eGFP coding sequence and SV40 3′UTR (Liu et al., 2013). The Lis1 cDNA [clone LD11219; obtained from the *Drosophila* Genomic Resource Center (DGRC; Bloomington, IN)] was amplified by PCR primers containing PmeI and AvrII restriction sites, which were used for cloning into the plasmid backbone. *Drosophila* S2R+ cells (low passage number of a stock obtained from DGRC) were cultured in Schneider's insect medium (Gibco-Life Technologies) with 10% fetal bovine serum (Labtech International) and 1× penicillin-streptomycin at 25°C. Cells were plated in an eight-well Nunc Lab-Tek chambered coverglass (Thermo Scientific) and transfected with Fugene HD (Promega) following the manufacturer's instructions. After 24 h, the transfection mix was removed by washing with Schneider's medium. The actin polymerisation inhibitor cytochalasin D (Sigma-Aldrich) was added to the medium at a final concentration of 1 μM for 4 h to induce process formation (Lu et al., 2013). 200 nM MitoTracker Deep Red (Molecular Probes-Life Technologies) was added to the medium for a further 30 min. After washing off the residual dye with Schneider's medium, cells were imaged at 25°C with a Zeiss 780 confocal microscope with a 40× C-Apochromat water-immersion objective. Image series were acquired from a single focal plane for 2–5 min with an acquisition rate of 0.5 frames/s (continuous imaging). Images were smoothened in ImageJ as described above before assembling Fig. 4G,H and Fig. S4G.

### Imaging Ca^2+^ responses in wing neurons

Flies overexpressing the Ca^2+^ sensor GCaMP6f (Chen et al., 2013) under the control of *Appl-Gal4* were mounted in a modified version of the imaging chamber described above. The wings were not entirely covered by a coverglass (Fig. S1A), but were covered in 10S halocarbon oil. Single presumptive mechanosensory bristles were touched with a pulled glass needle coupled to a Piezo micro-translational stage operated through PIMikroMove software (Physik Instrumente). Presumptive mechanosensory bristles were identified by their stereotypical morphology (Palka et al., 1979). Image series were recorded with the spinning disk imaging system with an acquisition time of 1 frame/s and exposure time of 0.3 s. The mean intensity of cell body fluorescence within a region of interest of fixed size was calculated in ImageJ after subtraction of background fluorescence.

### Statistics

Details of statistical evaluations are provided in the figure legends.
